# Electrochemical Mill Grinding of (TiB+TiC)/Ti6Al4V Composites Using Abrasive Tool with Bottom Outlet Holes

**DOI:** 10.3390/mi15121410

**Published:** 2024-11-23

**Authors:** Shen Niu, Kaiqiang Huang, Pingmei Ming, Ge Qin, Yansen Peng

**Affiliations:** 1 School of Mechanical and Power Engineering, Henan Polytechnic University, Jiaozuo 454003, China; hkq15638019737@163.com (K.H.); mingpingmei@163.com (P.M.); qinge@hpu.edu.cn (G.Q.); 13526468607@163.com (Y.P.); 2 Zhengzhou Institute for Advanced Research, Henan Polytechnic University, Zhengzhou 450018, China; 3 Henan International Joint Laboratory of Advanced Electronic Packaging Materials Precision Forming, Henan Polytechnic University, Jiaozuo 454003, China

**Keywords:** electrochemical mill grinding, (TiB+TiC)/Ti6Al4V composites, flatness, flow field simulation, surface quality

## Abstract

Difficult-to-cut titanium matrix composites (TMCs) are widely used in the aerospace, automotive, and defense sectors due to their excellent physical properties. Electrochemical mill grinding (ECMG) can achieve the processing effects of electrochemical milling and electrochemical grinding using the same tool, which has the potential to complete the rough and finish machining of TMCs in succession. However, in the rough machining stage, the bottom of the slot becomes concave due to the inevitable stray corrosion, leading to poor flatness, which increases the machining allowance for subsequent finish machining. In this paper, a bottom outlet hole layout of an abrasive tool with a diameter of 6 mm is proposed. Dynamic simulations demonstrate that the electrolyte flow rate in both side regions of the slot is significantly increased by the bottom outlet holes. The experimental results confirm that, compared with the tool without bottom outlet holes, a 61.2% reduction in the bottom flatness can be achieved when using the newly proposed tool during rough machining. After the finish machining, a slot with a width of 8 mm and a depth of 4.8 mm was obtained on the TMCs, which had a flat bottom and sidewall surface with good surface quality.

## 1. Introduction

Titanium matrix composites (TMCs) are a type of metal matrix composite composed of a titanium alloy matrix reinforced with strengthening phases such as TiB and TiC [1,2]. These composites retain the beneficial properties of titanium alloys, including their high strength and hardness, while the reinforcing phases further improve their specific strength, wear resistance, and thermal resistance [3,4]. Consequently, TMCs exhibit considerable potential for applications across the aerospace, automotive, marine, and defense sectors. However, the brittleness and hardness of the reinforcing phases and the low thermal conductivity of the titanium alloy matrix present significant challenges in the machining of TMCs, leading to significant tool wear and poor surface quality during conventional cutting processes [5,6]. Therefore, the processing of TMCs represents a current research focus.

Various non-traditional machining techniques based on different principles have been investigated for machine TMCs, including electrical discharge machining (EDM), laser additive manufacturing (LAM), and electrochemical (EC) milling. Zhang et al. [7] studied the influence of the processing parameters on EDM, and a slot with a surface roughness of Ra 0.9 μm was machined on TMCs. Wei et al. [8] obtained a cubic TMC sample with dimensions of 6 × 6 × 6 mm via LAM. Compared with EDM and LAM, the surfaces machined through EC milling are free from heat-affected zones, recast layers, and defects such as porosity and warpage deformation [9,10,11]. Li et al. [12,13] proposed the EC milling of TMCs using NaCl electrolyte, and a slot structure with a depth of 0.71 mm and a surface roughness of 4.281 μm was obtained. However, stray corrosion inevitably occurs during EC milling, which leads to poor accuracy and quality of the machined surface [12,13,14,15,16,17,18]. Thus, high-efficiency and high-quality machining of complex TMC structures continues to present a significant challenge.

Electrochemical mill grinding (ECMG) uses a rod-shaped tool embedded with abrasive particles as the cathode, employing a method similar to CNC milling. This technique combines electrochemical anodic dissolution with mechanical grinding to remove workpiece material. It offers high processing efficiency, good flexibility, low tool wear, high machining precision, and good surface quality, making it a promising approach for the efficient and precise machining of difficult-to-machine metal materials [19,20,21,22,23,24,25,26,27]. Initially, ECMG used an external nozzle to spray electrolyte onto the surface of a workpiece. Qu et al. [19] used a spherical abrasive tool with a diameter of 6 mm and a cutting depth of 0.5 mm to process Inconel 718. By optimizing the processing parameters, they enhanced the electrochemical dissolution during the material removal process, and a material removal rate of 10.41 mm^3^/min was attained. To further enhance the machining efficiency, an internal electrolyte spray approach was subsequently proposed, allowing for deeper cuts in a single pass. Li et al. [20] employed an abrasive tool with a diameter of 6 mm equipped with sidewall outlet holes and obtained a material removal rate of 44.44 mm^3^/min when machining Inconel 718 at a cutting depth of 3 mm. For an abrasive tool with a diameter of 10 mm and a cutting depth of 10 mm, Niu et al. [21,22,23] proposed various layouts for sidewall outlet holes, attaining material removal rates of 216.6 mm^3^/min for Inconel 718 and 248.3 mm^3^/min for Ti-6Al-4V during the rough machining stage.

During ECMG, some of the electrolytes sprayed from the sidewall outlet holes of the tool flow at high speed in the opposite direction to the feed movement over the machined surface, creating a conductive circuit between the tool and the machined surface. In the rough machining stage, the high voltage between the tool and the workpiece unavoidably leads to stray corrosion on the machined surface, resulting in poor surface flatness and quality. For an abrasive tool with a diameter of 10 mm, Niu et al. [21,22,23] further proposed an efficient and precise ECMG method. When using a high voltage, large cutting depth, and slow feed rate, the material removal primarily relies on electrochemical dissolution, allowing the rapid removal of most of the machining allowance. When a low voltage, small cutting depth, and fast feed rate are employed, the material removal mainly depends on grinding action, aiming to improve the flatness of the surface machined by the rough machining stage and reduce the surface roughness value. Compared with rough machining, after the finish machining stage, the bottom flatness and surface roughness (Ra) of Inconel 718 slots decreased by 90.6% and 88.3%, respectively [22]. For Ti-6Al-4V slots, the sidewall flatness and the surface roughness (Ra) decreased by 79.2% and 68%, respectively [23]. Consequently, ECMG can carry out both the rough and finish machining stages in the same workpiece using the same tool, thereby mitigating machining errors associated with tool changes and reducing the overall production cycle for components.

Surface flatness is an important technical requirement in the machining of components. Poor surface flatness in the rough machining stage can lead to a larger machining allowance for subsequent finish machining, resulting in a longer machining time. Niu et al. [25] found via electric field simulations that, when using an abrasive tool with a conductive end face, the electric charge distribution on the slot bottom gradually increases from the edges toward the center. According to Faraday’s law, the mass of material dissolved at the workpiece anode is directly proportional to the amount of charge passed. Consequently, during the rough machining stage of ECMG, material removal at the center of the slot bottom is significantly higher than that at the edges, leading to severe concavity on the bottom surface. At present, scholars tend to improve the flatness of the groove bottom by regulating the electric field distribution on the slot bottom surface. Li et al. [28] proposed an abrasive tool with a conical concave at the bottom at a diameter of 6 mm. Through alteration of the electric field distribution at the slot bottom, a 15% diminution in the maximum clearance at the machined slot bottom was attained. Niu et al. [25] proposed an end face insulation strategy for an abrasive tool with a diameter of 6 mm. By altering the electric charge distribution on the slot bottom through the insulated end face of the tool, the bottom flatness decreased by 58%, significantly improving the flatness during the rough machining stage. However, the tool with bottom insulation or a conical concave cannot be used for finish machining of the slot bottom. Moreover, the complex manufacturing process of the bottom insulation layer reduces the machining efficiency and precision of ECMG. Furthermore, there is limited research on TMCs in the realm of ECMG.

In this study, a layout of bottom outlet holes in an abrasive tool with a diameter of 6 mm is proposed to improve the surface flatness of TMCs during ECMG. Firstly, the influence of the bottom outlet holes on the electrolyte flow rate distribution across the bottom slot is investigated via dynamic flow field simulations. Subsequently, rough machining experiments are conducted to assess the effects of the bottom outlet holes’ layout on the sectional profiles of the slot. Ultimately, the newly proposed tool is employed to achieve rough and finish machining in succession for a slot on (TiB+TiC)/Ti6Al4V composites.

## 2. Materials and Methods

### 2.1. Design of the Tool with Bottom Outlet Holes

**Figure 1:**
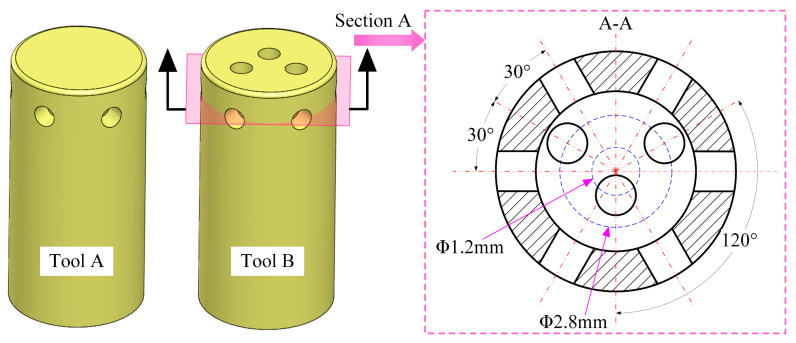
Design of the tool with bottom outlet holes.

The fundamental structure of the tool is illustrated in Figure 1. Tool A is a tube electrode with an outer diameter of 6 mm, a wall thickness of 1 mm, and a closed bottom. Six evenly distributed outlet holes with a diameter of 1 mm are located on the sidewall, and the centerlines of the holes are positioned 1.5 mm from the bottom face. A fillet with a radius of 0.2 mm is incorporated at the junction between the sidewall and the bottom face to enhance the uniformity of the flow field at the turns within the inter-electrode gap. Three outlet holes 1 mm in diameter were added to the bottom face of tool A, which is named tool B. Section A is defined as the plane where the centerlines of the sidewall outlet holes on tool B are situated, with a distance of 1.5 mm from section A to the bottom face. The cross-sectional view of section A reveals that the centers of the bottom outlet holes are arranged on a set of concentric circles on the bottom face of tool B. One bottom outlet hole is located on a circle with a diameter of 1.2 mm, while the other two are positioned on a circle with a diameter of 2.8 mm. The centerlines of the adjacent bottom outlet holes form an angle of 120°, and each centerline of the bottom outlet hole is spaced 30° apart from the centerlines of the adjacent sidewall outlet holes. The total area of the three bottom outlet holes on tool B constitutes only 9.6% of the total bottom face area, and thus the influence of the bottom outlet holes on the electric field within the gap between the workpiece surface and the bottom face of the tool can be regarded as negligible.

### 2.2. Experimental System and Arrangement

**Figure 2:**
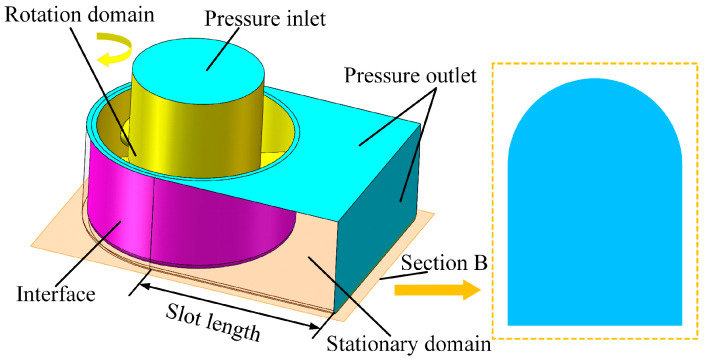
Geometric model for flow field simulation.

To analyze the effect of the bottom outlet holes’ layout on the electrolyte flow rate across the workpiece surface, a numerical simulation of the rotating flow field was carried out by employing the sliding mesh method. Figure 2 illustrates the geometric model employed for the dynamic flow field simulation. In the numerical simulation analysis, the rotating and stationary regions were in relative rotation, and the boundary interface between these two regions was interconnected through a mesh interface, thereby achieving flow field coupling. Section B was positioned between the bottom face of the tool and the workpiece surface, remaining parallel to the workpiece surface. The geometric parameters of the flow field model are defined as follows: a cutting depth of 3 mm, an inter-electrode gap of 0.3 mm, a tool height of 5 mm, a slot length of 6 mm, and the boundary interface at a distance of 0.15 mm from the outer surface of the tool. Unstructured tetrahedral meshes with an element size of 0.1 mm were employed for the discretization of the rotation domain and stationary domain. The boundary conditions for the numerical simulation are defined as follows. The inlet pressure was set to 0.5 MPa, the outlet pressure was established at atmospheric pressure, the rotational speed of the rotating region was 1000 rpm, the unsteady calculation time step was 0.00025 s, and the total number of time steps was 480, corresponding to a 720° rotation of the rotating region. Numerical simulation calculations were executed using the ANSYS FLUENT 15 finite element simulation module.

**Figure 3:**
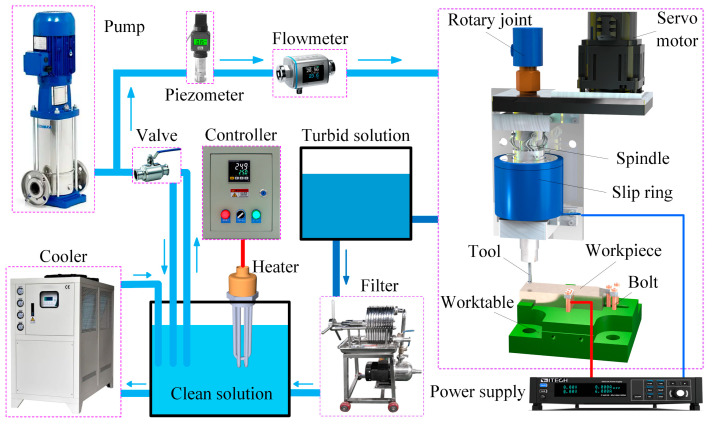
Schematic of the ECMG experimental system.

Figure 3 presents the schematic of the ECMG experimental system, including the spindle unit, power supply, and electrolyte circulation and filtration unit. The spindle unit employs a servo motor to drive the spindle for high-speed rotation through a synchronous belt mechanism. The spindle features a through-hole design, with the abrasive tool at its lower end. A rotary joint is positioned at the upper end of the spindle, facilitating the internal coolant supply during rotation. A slip ring located at the midpoint of the spindle delivers electrical current to the rotating tool. The workpiece is secured on a worktable, where a bolt is positioned at the clamping point. The slip ring and bolt are connected to the negative and positive poles of the power supply, respectively. The rotary joint is linked to the electrolyte circulation and filtration unit, which includes a pump, filter, and chiller, ensuring a stable supply of purified electrolytes to the machining area at the required temperature and pressure.

**Figure 4:**
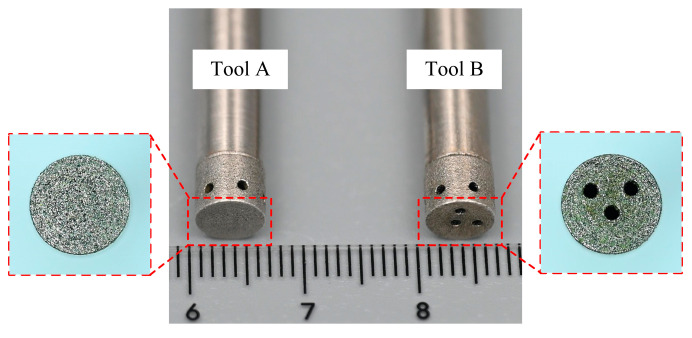
Photographs of tool A and tool B.

Figure 4 illustrates tool A and tool B, fabricated using the electroplating process. The material of the tool body was carbon steel, and the electroplating bonding agent was metallic nickel, with diamond particles 75–90 μm in size and a concentration of 8.8 carat/cm^3^. The thickness and height of the electroplated nickel layer were 0.1 mm and 5 mm, respectively. The (TiB+TiC)/Ti6Al4V composites were provided by Zhejiang Jiatai Metal Technology Co., Ltd. (Wenzhou, China). The matrix material was Ti6Al4V titanium alloy, and the reinforcing phase was TiB and TiC. The density of the (TiB+TiC)/Ti6Al4V composites was approximately 4.52 g/cm^3^, and the chemical composition of the material is presented in Table 1. The electrolytes used were aqueous NaNO_3_ electrolytes with a mass fraction of 10% at a temperature of 30 °C.

The ECMG of the slot structure for the (TiB+TiC)/Ti6Al4V consisted of two stages: the rough machining stage and the finish machining stage. During the rough machining stage, the cutting depth per pass was set to 3 mm. Initially, a length of 3.2 mm was machined from the sidewall of the workpiece at a feed rate of 0.7 mm/min, followed by an additional 19.8 mm machined at a feed rate of 1.5 mm/min. The process parameters of the rough machining stage are detailed in Table 2. In the finish machining stage, the cutting depth for each pass was set to 25 μm, and the feed rate was set to 30 mm/min. The bottom surface of the slot was machined layer by layer 1.5 mm at a time, and then the left and right sidewall of the slot were machined layer by layer 1 mm at a time, respectively. The process parameters of the finish machining stage are exhibited in Table 2. Each experiment was repeated three times to ensure reproducibility.

The workpieces were cleaned ultrasonically and dried both before and after the experiments. Then, the weight of the workpiece was measured using an electronic balance with an accuracy of 0.01 g. The machined surface contour was determined at a distance of 10 mm from the slot entrance using a super depth-of-field 3D microscope (VHX-600, KEYENCE, Osaka, Japan). The coordinates of the points on the machined surface profile of the slot were measured by a coordinate measuring machine (ZEISS CONTURA, Oberkochen, Germany). In addition, a roughness meter (Perthometer M1, Mahr, Göttingen, Germany) was used to measure the surface roughness of the side and bottom surfaces of the slot. The surface morphologies of both the side and bottom surfaces of the machined slot were analyzed via a scanning electron microscope (S-3400, Hitachi, Tokyo, Japan).

## 3. Results and Discussion

### 3.1. Flow Field Simulation Analysis

**Figure 5:**
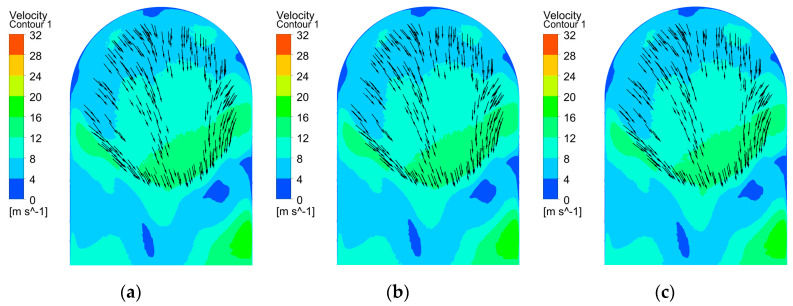
Distribution of electrolyte flow velocity on section B using tool A at different rotational angles: (**a**) 420°; (**b**) 540°; and (**c**) 660°.

Figure 5 presents the distribution of the electrolyte flow velocity on section B at different rotational angles when using tool A. The results indicate that the electrolyte flow velocity across the workpiece surface remained relatively uniform at different rotational angles, and the flow rate in the central region was higher than those in the side regions. As shown in Figure 5a, the velocity vectors indicate that the electrolyte entering the bottom gap flowed toward the central region due to the geometry of the transition surface of the workpiece. Throughout the complete rotation of tool A, the flow rates for both side regions of the bottom surface were consistently lower than that in the central region. The decreased electrolyte velocity restricted the transportation of machined products, thereby reducing the conductivity of the electrolyte and material removal rate. As a result, when an electric field was applied, the material dissolution rate in the central region of the workpiece surface was substantially higher than those in both side regions, leading to a concave surface profile at the bottom of the workpiece.

**Figure 6:**
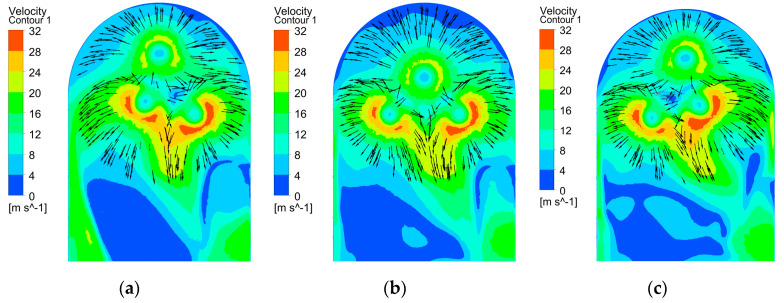
Distribution of electrolyte flow velocity on section B using tool B at different rotational angles: (**a**) 420°; (**b**) 540°; and (**c**) 660°.

Figure 6 depicts the distribution of the electrolyte flow velocity on section B at various rotational angles when employing tool B. It is evident that at each rotational angle, the electrolyte flow velocities in both side regions of the workpiece surface experienced a significant increase, while the flow velocity in the central region diminished. As shown in Figure 6a, the velocity vectors illustrate that the electrolytes ejected from the sidewall outlet holes collided with the electrolytes released from the bottom outlet holes when they entered the bottom gap. This interaction caused the electrolytes which flowed toward the central region to be redirected toward both side regions. In addition, the electrolytes ejected from the adjacent end injection holes flowed toward the edge area to flow at a high velocity after the collision. Consequently, in comparison with tool A, the electrolyte flow rate in for both side regions of the bottom surface of the workpiece was significantly enhanced when using tool B, which is attributed to the regulating effect of the bottom outlet holes. This increase in electrolyte flow rate enhanced the electrolyte conductivity and material dissolution rates in both side regions, thereby reducing the disparity in material removal between the central and both side regions of the workpiece surface, which can theoretically mitigate the concavity of the workpiece bottom.

### 3.2. Machining with ECMG

#### 3.2.1. Rough Machining with ECMG

**Figure 7:**
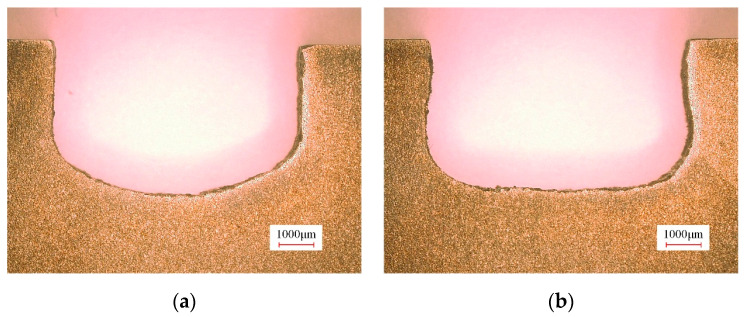
Machined surface profiles of slots processed with tool A and tool B: (**a**) tool A and (**b**) tool B.

During the rough machining stage, the machined surface profiles of the slots of the (TiB+TiC)/Ti6Al4V composites machined with tool A and tool B are shown in Figure 7. It can be observed that the bottom surface profile of the slot machined with tool A exhibited a concave shape, while the contour of the slot machined with tool B was flatter. The flow field simulation analysis indicates that the electrolyte flow rates for both side regions of the bottom surface of the workpiece were significantly enhanced when using tool B, which is attributed to the regulating effect of the bottom outlet holes. Consequently, compared with tool A, when using tool B, the material removal rates for both side regions of the bottom surface were significantly enhanced, which effectively improved the flatness of the slot bottom. However, an increase in the electrolyte flow velocities for both side regions of the slot bottom will inevitably lead to an increase in the amount of material removed from the trench root, thereby leading to a more pronounced rounded transition at the junction of the slot bottom and sidewall surface and contributing to a deterioration in the sidewall flatness of the slot.

**Figure 8:**
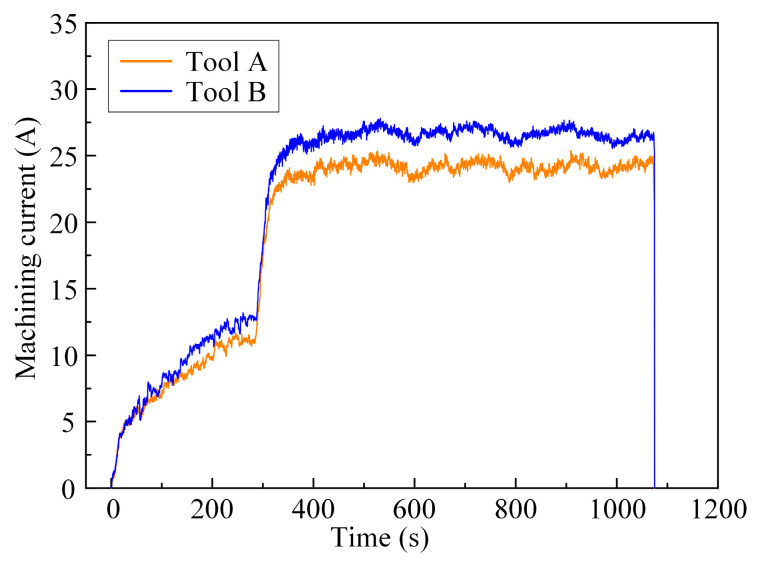
Machining current signal during processing.

Figure 8 shows the evolution of the current over time during the entire machining process using tool A and tool B. When the initial feed rate was 0.7 mm/min, the current increased slowly as the abrasive tool began to enter the workpiece. When the feed rate reached 1.5 mm/min, the current rose rapidly and then tended to stabilize, which was caused by a sharp reduction in the machining clearance. Throughout the entire machining process, the machining current when using tool B was almost always greater than that when using tool A. During the stable stage of the current, the current was approximately 26.73 A when using tool B, while it was approximately 24.21 A when using tool A. The reason for this difference lies in the fact that three bottom outlet holes were added to tool B. Consequently, during the machining process with tool B, the flow rate of the electrolytes was greater than that with tool A. The electrolyte flow rate for tool A was 6.12 L min^−1^, while that for tool B was 8.26 L min^−1^. Therefore, compared with tool A, when using tool B for machining, the flow rate of the electrolytes in the gap between the bottom of the groove and the end face of the tool significantly increased, the velocity of product removal was enhanced, the electrical conductivity of the electrolyte in the machining area increased, and finally, the machining current increased.

**Figure 9:**
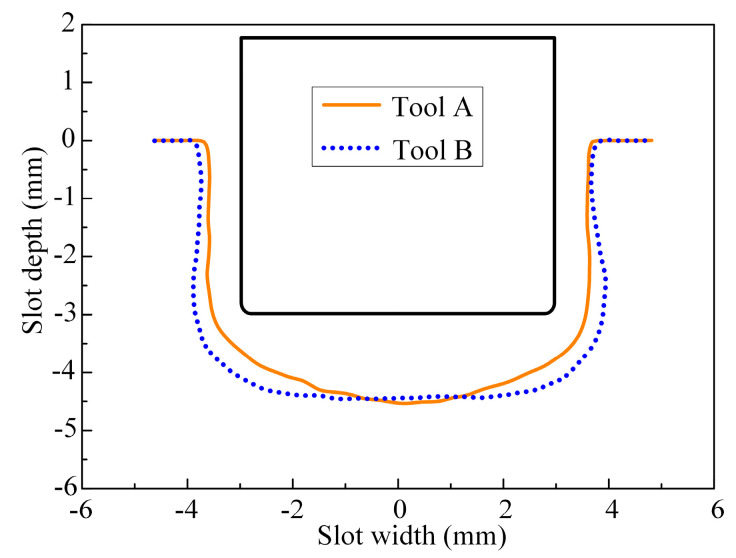
Three-dimensional coordinate measurement curves of machined surface profiles of slots.

Figure 9 shows the three-coordinate measurement curves of the machined surface profiles of the slots. It can be observed that the slot depths in the central area of the bottom surface were quite similar for both tool A and tool B. Compared with tool A, the slot depths in both side regions of the bottom surface were significantly enhanced when using tool B, resulting in a flatter bottom profile. Nevertheless, the transition fillet radius at the junction of the bottom and sidewall surfaces was larger when using tool B, which increased the disparity in slot width between the root and top of the sidewall, thereby diminishing the sidewall flatness of the slot.

**Figure 10:**
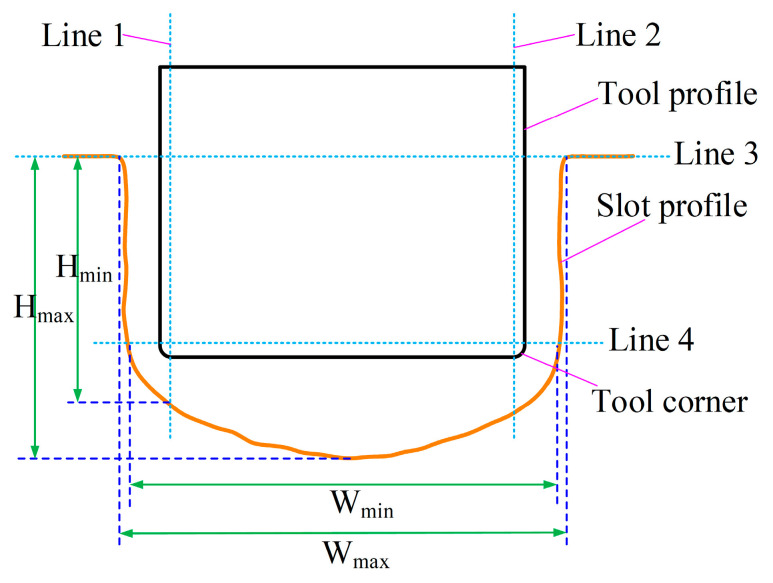
Measurement method for dimensions and flatness of machined surface profiles of slots.

To reflect the influence of the bottom outlet holes on the dimensions and flatness of the machined surface contour of the slot, the maximum slot width, maximum slot depth, bottom flatness, and sidewall flatness were defined. As illustrated in Figure 10, in order to mitigate the influence of the rounded corners of the tool, the maximum and minimum distances of the measurement points located between lines 1 and 2 and line 3 on the machined surface contour of the slot were defined as the maximum and minimum slot depths, respectively. The maximum and minimum horizontal distances between the measurement points situated between lines 3 and 4 on the machined surface contour of the slot were defined as the maximum and minimum slot widths, respectively. Concurrently, the difference between the maximum and minimum slot depths was identified as the bottom flatness, while half the difference between the maximum and minimum slot widths was designated as the sidewall flatness. Thus, the bottom flatness F_b_ and sidewall flatness F_s_ are given by

F_b_ = H_max_ − H_min_, (1)

Fs = (W_max_ − W_min_)/2, (2)

where H_max_ and H_min_ represent the maximum and minimum slot depths, respectively, and W_max_ and W_min_ denote the maximum and minimum slot widths, respectively (refer to Figure 10).

**Figure 11:**
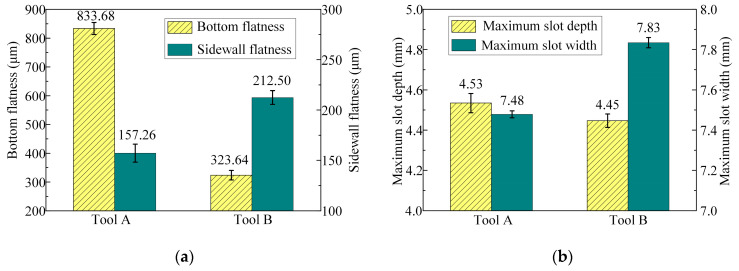
Dimensions and flatness of the machined surface of the slots machined with tool A and tool B: (**a**) bottom flatness and sidewall flatness and (**b**) the maximum depth and width.

Figure 11 illustrates the bottom flatness, sidewall flatness, and maximum slot depth and width of the machined surface contours of the slots processed with tools A and B. The results demonstrate that, compared with tool A, the bottom flatness was reduced from 833.68 μm to 323.64 μm when using tool B, with a decrease of 61.2%. The maximum slot depth decreased from 4.53 mm to 4.45 mm, which could be negligible. In contrast, the sidewall flatness increased from 157.26 μm to 212.5 μm, with an increase of 35.13%, while the maximum slot width rose from 7.48 mm to 7.83 mm. These results indicate that the layout of the bottom outlet holes significantly mitigated the material removal disparity between both side regions and the central region of the bottom surface, while the material removal difference between the root and the top of the sidewall surface increased. Consequently, the bottom flatness was effectively improved, while the sidewall flatness deteriorated. Overall, the reduction in bottom flatness was considerably greater than the increase in sidewall flatness. Therefore, the machining allowance of the finish machining stage can be significantly reduced when using tool B, which is more suitable for the rough machining stage in ECMG.

#### 3.2.2. Finish Machining with ECMG

**Figure 12:**
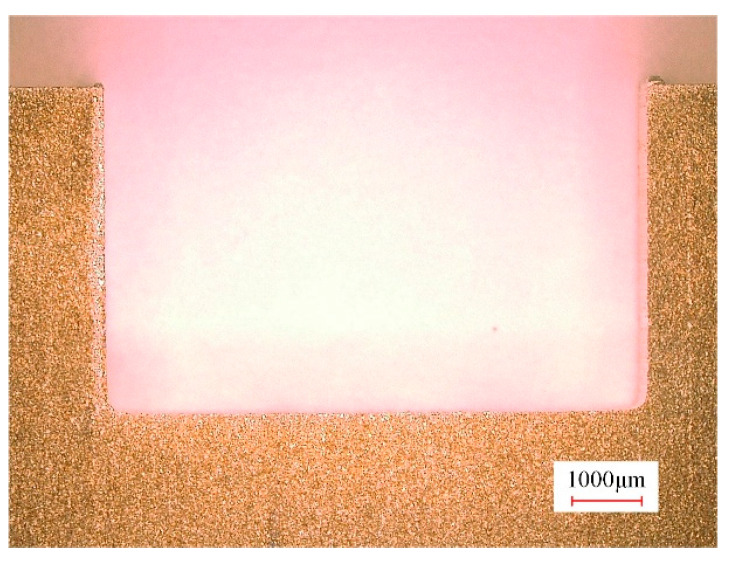
Machined surface profile of the slot using tool B after finish machining.

**Figure 13:**
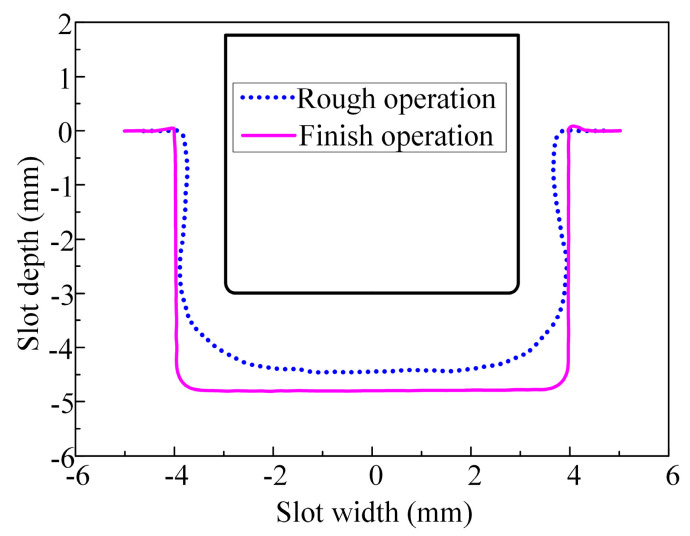
Three-dimensional coordinate measurement curves of machined surface profiles for rough and finish machining of slots.

The machined surface profile of the slot obtained during the finish machining stage with tool B is presented in Figure 12. Measurements revealed that the total material removal volume during both the rough and finish machining stages was 847.35 mm^3^, with 692.48 mm^3^ removed in the rough machining stage, accounting for 81.7% of the total material removed. This indicates that during the rough machining stage, ECMG was in an overcutting state, and the majority of the machining allowance for the slot was removed. Figure 13 further presents a comparison of the three-coordinate measurement curves of the machined surface profiles for both the rough and finish machining stages. It can be observed that after finish machining, the bottom and sidewall surfaces of the slot profiles were smooth, with a slightly rounded corner at the transition between the bottom and sidewall surfaces. The results indicate that ECMG can achieve a zero-overcut state during the finish machining stage, where the motion trajectory of the diamond grains on the bottom and sidewall surface accurately reflects the contour of the bottom and side surfaces of the slot.

**Figure 14:**
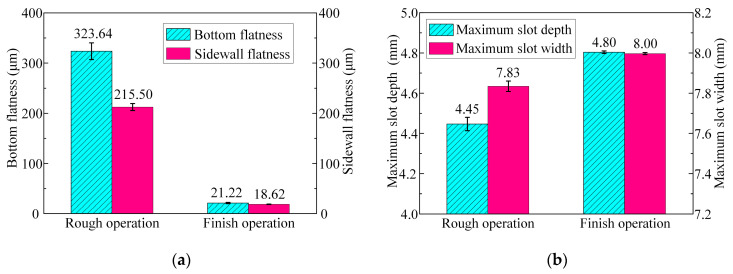
Dimensions and flatness of the machined surface of the slots machined by rough and finish operations: (**a**) bottom flatness and sidewall flatness and (**b**) the maximum depth and width.

Figure 14 shows the dimensions and flatness of the slots after the rough and finish machining stages. In comparison with the rough machining stage, after the finish machining stage, the bottom flatness of the slot decreased from 323.64 ± 16.66 μm to 21.22 ± 1.28 μm, with a decrease of 93.4%. The sidewall flatness decreased from 212.5 ± 6.9 μm to 18.62 ± 0.51 μm, with a decrease of 91.2%. The maximum slot depth increased from 4.45 ± 0.033 mm to 4.8 ± 0.006 mm, and the maximum slot width increased from 7.83 ± 0.026 mm to 8 ± 0.005 mm. The standard deviations of the slot depth and width were reduced by 81.3% and 80.4%, respectively. These experimental results substantiate that the bottom flatness, sidewall flatness, and consistency of the slot depth and width were significantly improved following the finish machining stage.

**Figure 15:**
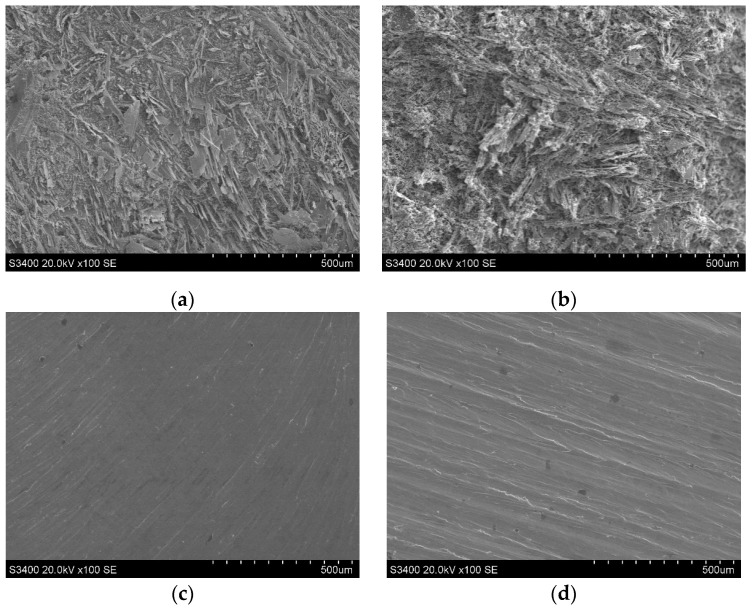
Surface morphologies of bottom and sidewall surfaces of rough and finish machined slots: (**a**) the rough machined bottom; (**b**) the rough machined sidewall; (**c**) the finish machined bottom; and (**d**) the finish machined sidewall.

Figure 15 illustrates the morphology of the bottom and sidewall surfaces of the slots after the rough and finish machining stages. It can be seen that the bottom and sidewall surfaces of the slot after the rough machining stage displayed severe stray corrosion, characterized by uneven dissolution of the base material which exposed numerous fibrous reinforcement phases, resulting in irregular pits. In contrast, the bottom and sidewall surfaces of the slots after the finish machining stage exhibited a uniform and smooth morphology, with raised streaks discernible on the surface. This observation indicates that the material removal during the finish machining stage is predominantly reliant on grinding, with no stray corrosion on the machined surfaces. However, the grinding marks on the side surface were more pronounced compared with those on the bottom surface. This disparity arises from the fact that the finish machining of the slot sidewall is conducted using the circumference of the tool, while the bottom surface is machined using the end face of the tool. The latter method permits a greater number of diamond abrasives entering the grinding zone throughout the machining process, and the distribution of abrasives is more uniform. Consequently, the height of the raised features formed on the bottom surface after abrasive cutting was significantly lower than that on the sidewall surface, leading to shallower scratches and a smoother overall surface.

**Figure 16:**
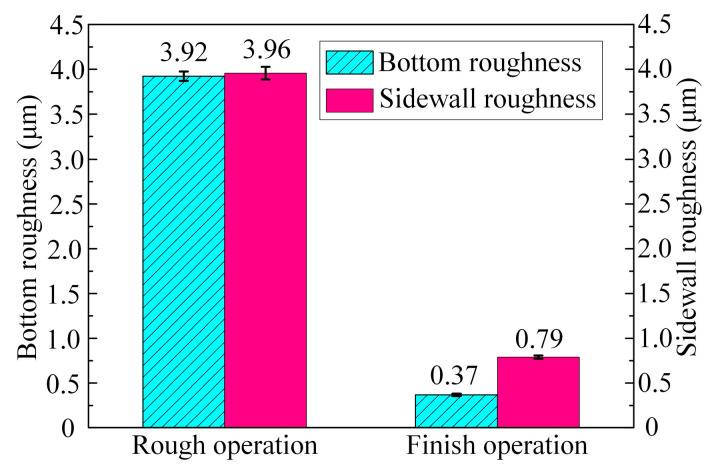
Surface roughness of bottom and sidewall surfaces of rough and finish machining slots.

**Figure 17:**
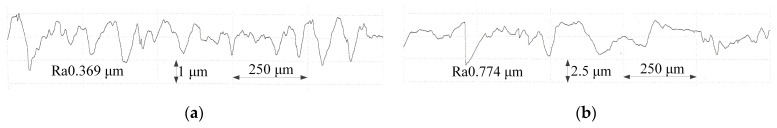
Surface roughness of the finish machined surface: (**a**) bottom surface and (**b**) sidewall surface.

Figure 16 presents a comparison of the surface roughness of the bottom and sidewall surfaces of the slots following the rough and finish machining stages. Compared with the rough machining stage, after the finish machining stage, the bottom surface roughness of the slot decreased from Ra 3.92 ± 0.053 μm to 0.37 ± 0.014 μm, with a decrease of 90.6%, while the sidewall surface roughness decreased from an Ra of 3.96 ± 0.071 μm to 0.79 ± 0.019 μm, with a decrease of 80.1%. The surface roughness of the finely machined surface is shown in Figure 17. It can be seen that, compared with the sidewall surface of the slot, the bottom surface of the slot was flatter and had a smaller height difference between the peaks and valleys. These experimental results confirm that the surface quality of both the bottom and side surfaces of the slots was significantly enhanced after finish machining. Therefore, the newly proposed tool can complete rough and finish machining in succession on (TiB+TiC)/Ti6Al4V composites through ECMG.

## 4. Conclusions

To enhance the surface flatness of ECMG for (TiB+TiC)/Ti6Al4V composite materials, a strategic layout of bottom outlet holes was proposed for a cylindrical abrasive tool with a diameter of 6 mm. Through dynamic flow field simulations, the influence of the bottom outlet holes’ layout on the electrolyte flow distribution across slot bottom surfaces was analyzed. The influence of the bottom outlet holes on the section profiles of the slots was investigated with a rough machining experiment. Rough and finish machining in succession was accomplished for the (TiB+TiC)/Ti6Al4V composite slots using a newly proposed tool. The results led to the following conclusions:

1. The results of the dynamic flow field simulation indicate that, in comparison with the tool without bottom outlet holes, the electrolyte flow rates in both side regions of the bottom surface can be significantly increased with a reasonable layout for the bottom outlet holes, which can improve the uniformity of the flow field in the bottom surface.
2. The results of the rough machining experiment demonstrate that, compared with the tool without bottom outlet holes, a reasonable layout of bottom outlet holes significantly improves the flatness of the machined bottom surface. This optimization results in a 61.2% reduction in the bottom flatness of the slot, thereby reducing the machining allowance for finish machining.
3. In comparison with rough machining, both the surface flatness and quality of the slots significantly improved after the finish machining. The bottom flatness and sidewall flatness were reduced by 93.4% and 91.2%, respectively. Furthermore, the surface roughness of the bottom and sidewall surfaces was reduced by 90.6% and 80.1%, respectively.
4. A slot was obtained in the (TiB+TiC)/Ti6Al4V composites via ECMG using the newly proposed tool, which has a width of 8 mm and a depth of 4.8 mm. The bottom flatness and sidewall flatness were 21.22 μm and 18.62 μm, respectively. In addition, the bottom and sidewall surface roughness were 0.37 μm and 0.79 μm, respectively.

## Figures and Tables

**Table 1:** Chemical compositions of (TiB+TiC)/Ti6Al4V Composites.

**Element**	Ti	V	Fe	Al	H	O	N	C	TiB	TiC
**Content (wt%)**	81.996	3.671	0.275	5.507	0.014	0.184	0.046	0.092	6.470	1.745

**Table 2:** The process parameters of the rough and finish machining stages.

Parameter	Rough Machining	Finish Machining
Applied voltage	25 V	2 V
Depth of cut	3 mm	25 μm
Electrolyte inlet pressure	0.5 MPa	0.2 MPa
Rotation speed	1000 rpm	1000 rpm

## Data Availability

The original contributions presented in the study are included in the article, further inquiries can be directed to the corresponding author.
